# Resting state fMRI connectivity of amygdala and hippocampus in women with breast cancer prior to chemotherapy

**DOI:** 10.1192/j.eurpsy.2021.1092

**Published:** 2021-08-13

**Authors:** A. D’Imperio, C. Sicuso, M. Fiorelli, C. Mainero

**Affiliations:** 1 Department Of Human Neuroscience, Sapienza University of Rome, Roma, Italy; 2 Department Of Radiology, Humanitas Clinical ad Research Center, Rozzano, Italy; 3 Athinoula A.martinos Center For Biomedical Imaging, Massachuttes General Hospital, Charlestown, United States of America

**Keywords:** Resting sate fMRI, amygdala and hippocampus connectivity, PAOFI-Memory, subjective cognitive impairment

## Abstract

**Introduction:**

Cognitive complaints and psychological distress are common in oncologic patients, in particular many studies have focused on women with breast cancer.Patients presenting the phenomenon of “chemofog” show changes after chemotherapy with regard to memory and emotional regulaiton.

**Objectives:**

To explore brain connectivity prior to chemotherapy that nevertheless,is understudied.

**Methods:**

We used fMRI to investigate the resting state connectivity in 24 patients before chemotherapy and 15 controls.Patients were assessed with self-administered questionnaires,such as the Patient’s Assessment of Own Functioning Inventory (PAOFI) that quantifies the decrease in perceived functioning in memory, language and problem solving (Image 1).We used a preliminary structural analysis in order to choose which neuropsychological test was affected in correlation with a significant anatomical volume alteration,as showed in the p-value table.Therefore, patients were ranked and divided into two group of “Impaired vs Preserved”, measured using the median of the questionnaire results.Higher scores indicate a poor cognitive self-perceived performance.

**Figure:**
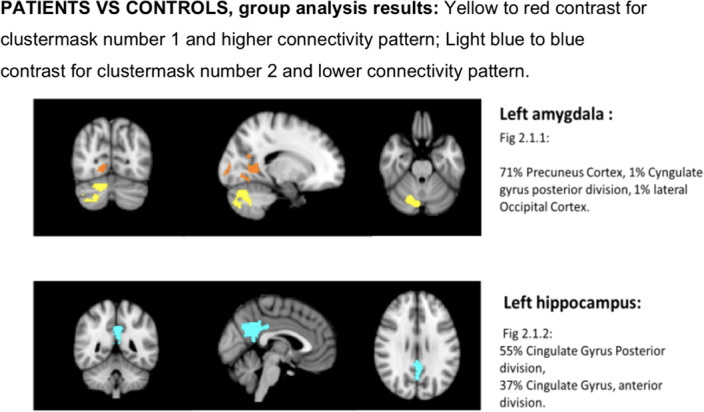

**Figure:**
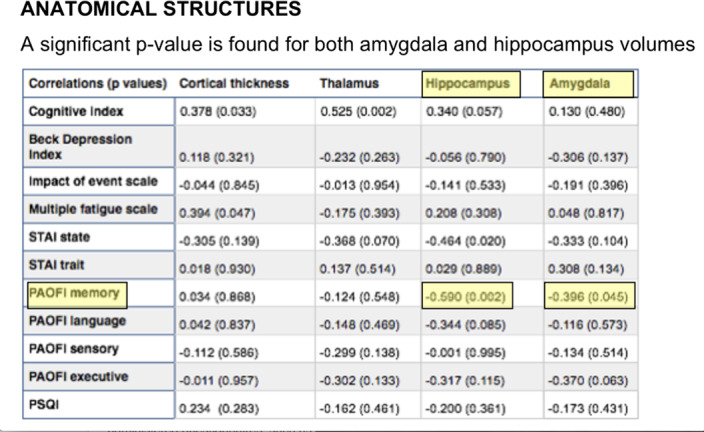

**Results:**

Connectivity was altered in amygdala and hippocampus, in the subgroup of patients with higher subjective cognitive complaints i.e with a high PAOFI Memory score.More specifically, we found an association between memory impairment and the increase of the resting state connectivity of both right structures, as opposed to a reduction in left amygdala (Image 3).

**Figure:**
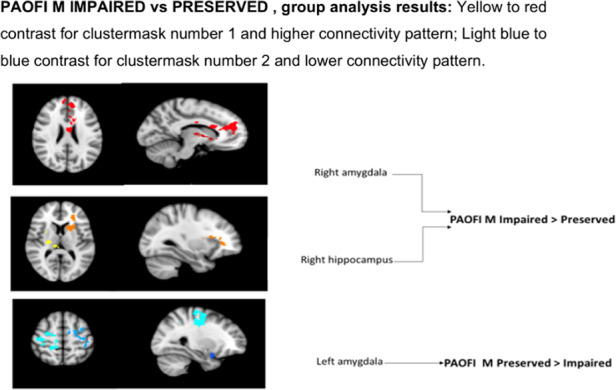

**Conclusions:**

These findings may suggest a potential effect on brain functional connectivity of the psychological awareness and stress of cancer itself. We found connectivity alterations for both amygdala and hippocampus, two structures belonging to the limbic system, that is involved in the interplay between cognition and emotions, such as anxiety and fear.

